# Voice activated remote monitoring technology for heart failure patients: Study design, feasibility and observations from a pilot randomized control trial

**DOI:** 10.1371/journal.pone.0267794

**Published:** 2022-05-06

**Authors:** Nawar Shara, Margret V. Bjarnadottir, Noor Falah, Jiling Chou, Hasan S. Alqutri, Federico M. Asch, Kelley M. Anderson, Sonita S. Bennett, Alexander Kuhn, Becky Montalvo, Osirelis Sanchez, Amy Loveland, Selma F. Mohammed

**Affiliations:** 1 MedStar Health Research Institute, Hyattsville, MD, United States of America; 2 Georgetown University, Washington, DC, United States of America; 3 Georgetown-Howard Universities Center for Clinical and Translational Science, Washington, DC, United States of America; 4 Center for Health Information and Decision Systems, University of Maryland, College Park, MD, United States of America; 5 MedStar Health National Center for Human Factors in Healthcare, MedStar Health Research Institute, Hyattsville, MD, United States of America; 6 Creighton University, Omaha, NE, United States of America; Universita degli Studi Magna Graecia di Catanzaro, ITALY

## Abstract

**Background:**

Heart failure (HF) is a serious health condition, associated with high health care costs, and poor outcomes. Patient empowerment and self-care are a key component of successful HF management. The emergence of telehealth may enable providers to remotely monitor patients’ statuses, support adherence to medical guidelines, improve patient wellbeing, and promote daily awareness of overall patients’ health.

**Objective:**

To assess the feasibility of a voice activated technology for monitoring of HF patients, and its impact on HF clinical outcomes and health care utilization.

**Methods:**

We conducted a randomized clinical trial; ambulatory HF patients were randomized to voice activated technology or standard of care (SOC) for 90 days. The system developed for this study monitored patient symptoms using a daily survey and alerted healthcare providers of pre-determined reported symptoms of worsening HF. We used summary statistics and descriptive visualizations to study the alerts generated by the technology and to healthcare utilization outcomes.

**Results:**

The average age of patients was 54 years, the majority were Black and 45% were women. Almost all participants had an annual income below $50,000. Baseline characteristics were not statistically significantly different between the two arms. The technical infrastructure was successfully set up and two thirds of the invited study participants interacted with the technology. Patients reported favorable perception and high comfort level with the use of voice activated technology. The responses from the participants varied widely and higher perceived symptom burden was not associated with hospitalization on qualitative assessment of the data visualization plot. Among patients randomized to the voice activated technology arm, there was one HF emergency department (ED) visit and 2 HF hospitalizations; there were no events in the SOC arm.

**Conclusions:**

This study demonstrates the feasibility of remote symptom monitoring of HF patients using voice activated technology. The varying HF severity and the wide range of patient responses to the technology indicate that personalized technological approaches are needed to capture the full benefit of the technology. The differences in health care utilization between the two arms call for further study into the impact of remote monitoring on health care utilization and patients’ wellbeing.

## Introduction

Heart failure (HF) is a common cardiovascular disease associated with serious health events and high health care costs. The foundation of HF disease management is medications and lifestyle changes. Such lifestyle changes include exercise, diet [1], and daily monitoring for symptoms such as shortness of breath, sudden weight gain, or swelling in the ankles that may indicate worsening HF severity [2].

Successful management of HF is heavily dependent on patient self-management in coordination with their healthcare provider. As such, patients can benefit from the advancement in remote monitoring technologies to support care coordination and management, a trend which has only accelerated during the COVID-19 pandemic. Technologies that help patients monitor their condition and send frequent updates to their provider can be particularly useful for the management of chronic conditions including HF, where routine monitoring is crucial, and prompt medical intervention can make a significant difference in outcomes.

Several studies examined different telehealth approaches for HF patients. A 2015 review included 41 studies of at-home devices and structured telephone support for HF patients. This review concluded utilizing at-home technology or telephone support for patients with HF can decrease the risk of HF related hospitalizations [3]. With the increased availability of reliable internet access, several internet-based technologies have been developed. Examples include a 2010 study of a web-interface that allows patients to log in and enter their daily symptoms, which in turn allows nurses to monitor changes in symptom severity [4]. A 2011 study used an Xbox gaming platform to allow HF patients to answer multiple-choice questions regarding their symptoms and read information both about their condition as well as their self-care [5]. More recently, tablets connected to a weight scale and a wrist blood pressure monitor have been used to send daily readings to healthcare providers [6]. Several recent studies assessed the use of smartphone apps that enabled HF patients to submit information about daily symptoms and vital signs, and receive feedback about their health [7–13]. A 2020 meta-analysis concluded utilizing telehealth tools had a positive effect on the lives of patients with HF [14].

In this paper, we report on a clinical trial using voice interface technology with the goal of empowering HF patients to better manage their own health. Voice activated technology enables a user to interact with technology devices using vocal prompts (as opposed to pressing buttons or using a keyboard) and is therefore more accessible to a larger group of patients than devices using traditional technology, including patients with vision impediments and those with impaired fine-motor skills. So far, voice recognition technologies have been incorporated into telehealth systems via automated calls [15–18], for example by introducing weekly interactive calls for HF patients support [17]. In the present study, we utilize voice activated technology embedded in smart devices, to support remote patient monitoring. The voice interface allowed patients to set medication or wellness reminders, offered real time access to health care information, and provided the patient’s care team with clinical alerts.

The study has two aims: first, we designed and tested a customized and interactive chronic HF-specific functionality (skill kit) within a voice activated technology (delivered in an Amazon’s Echo Dot) as a tool for patient self-management at home. Second, we studied the impact of this technology on clinical outcomes and health care utilization

## Methods

### Enrollment and study procedures

**Fig 1:**
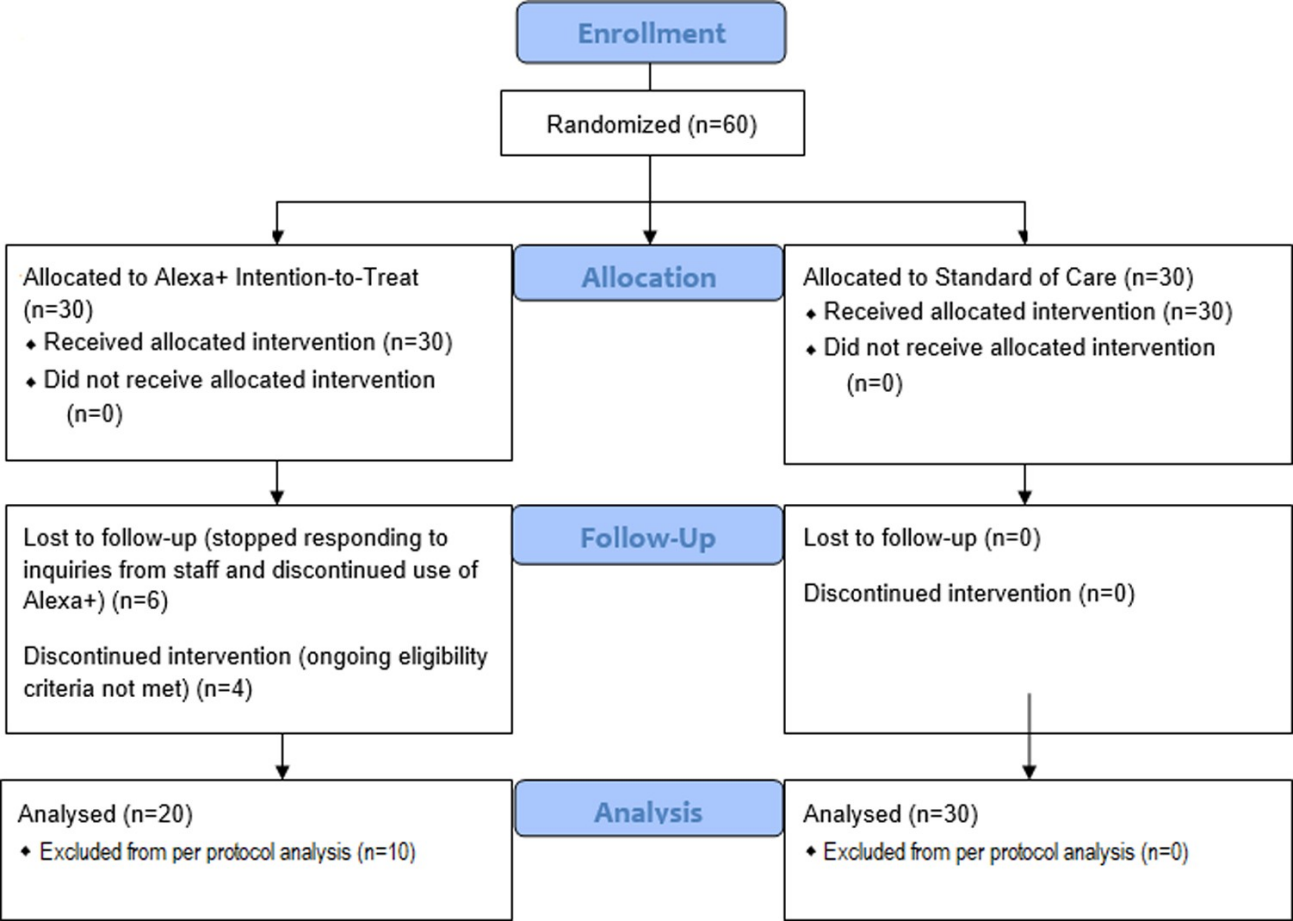
CONSORT diagram.

Patients were recruited from the MedStar Health Heart and Vascular Institute, Washington, DC from December 2018 through March of 2019 (Fig 1). The Heart and Vascular Institute HF clinic patient schedule and Electronic Health Records (EHR) were reviewed daily for patients meeting study inclusion criteria. Eligible patients were approached by the study coordinators after their scheduled visit was completed. The number of patients assessed for eligibility and reasons for exclusion were not collected. Participation was voluntary, and participants were not compensated monetarily; however, all patients received an Alexa device after the study. All patients were 18 or older, had chronic HF, and lived in a home with Wi-Fi access. Exclusion criteria included prior heart transplants and ventricular assist devices (VAD). All participants were fully informed of the study logistics as well as potential risks and benefits and have signed an informed consent document before participation in the trial.

The study consisted of sixty participants randomized 1:1 into two arms: the Alexa+ arm and the standard of care (SOC) arm. Patients were randomly allocated into these arms, random allocations were generated in seven blocks, each of size 10, using the R package ‘blockrand’. This process provided sealed envelopes that stated either “Alexa+” or “Standard of Care”. For each patient, the coordinator opened one of these envelopes and assigned the participant to the designated arm. This resulted in 30 patients in the Alexa+ arm and 30 in the SOC arm. Patients in the treatment arm received an Echo Dot device, a smart speaker that uses Amazon’s virtual assistant AI technology, preloaded with the Alexa+ HF questioner. An Amazon account was created for each patient in the Alexa+ arm so they could access the voice interface at home. The devices were set up and every participant underwent an in-person training on how to use the Alexa+ voice interface and how to respond to their first daily questionnaire. This proper device function and patients’ ability to use the device.

The Alexa+ patients’ answers in their first questionnaire established their baseline, which provided a basis for comparison for their subsequent survey answers or changes in condition. Patients in this study arm were asked to complete the survey daily for 90 days. The results were viewed daily by the study nurse, who evaluated each patient’s condition and compared it to their baseline. The study coordinator reviewed overall participation, and every three weeks, called participants who had not been completing the daily surveys. During these calls, the coordinator checked on the patient’s status and encouraged participation by reemphasizing the importance of the study and providing more training or information on device use if needed. Some patients had periods of non-response due to travel and informed the study coordinator of these plans in advance.

This study was approved by the Georgetown-Howard Universities Center for Clinical and Translational Science Institutional Review Board, and all participants in this study signed a written Informed Consent for Clinical Research.

### Technology development

To develop the voice interface and the capabilities to capture, record and report patients’ responses, we used the Alexa Skills Kit (ASK), which is a coding interface (consisting of APIs, tools and documentation) that allows developers to create applications that use Alexa’s voice and language technology. In this paper, we refer to the voice interface, and the application that captures, records and transmits patient’s answers as Alexa+.

A daily questionnaire was developed to capture adherence to self-care guidelines, and HF medications and reporting symptoms of worsening HF. The answers were captured and transferred to a Tableau dashboard (developed for this study) that was viewed by the study team. Answers were color-coded in accordance with the level of patient health risk they represent, and these “flags” triggered alerts that notified the provider of the patient’s responses and possible notification to the HF care team. The color-coded dashboard display for the study team was designed to summarize patients’ well-being and risk factors, a design informed by previous studies [4, 19, 20].

The questionnaire was based on previous studies related to the use of action plans to manage self-care and encourage symptom recognition among HF patients [7, 21, 22] along with literature on telehealth systems use in HF management [19, 23]. The survey was comprised of three sets of yes/no questions about a patient’s self-care activities and any signs or symptoms that could indicate a worsening of their HF. (See S1 Table for full script) The initial questions focus on adherence to self-care, then the questions enquire about increasingly severe HF symptoms. When a study participant answers an adherence question (asking whether patients have been weighing themselves, taking their medication, and avoiding high-sodium food), a non-adherence answer raises a blue flag. Two additional questions about medication adherence are only asked if the preceding question on medication adherence indicates non-adherence. For the mild HF symptom questions (swollen ankles, a cough, or shortness of breath with activity), a positive answer raises an orange flag. For the severe HF symptom questions (weight gain, or shortness of breath while resting/sleeping), a positive answer raises a red flag. Once an answer has been given, Alexa responds indicating the answer has been received, and the next question is asked. Once the patient completes the survey, the voice interface summarizes their responses for them and provides health and/or compliance information based on their specific responses. For example, if the patient did not weigh themselves, the summary includes a recommendation for the patient to do so.

**Fig 2:**
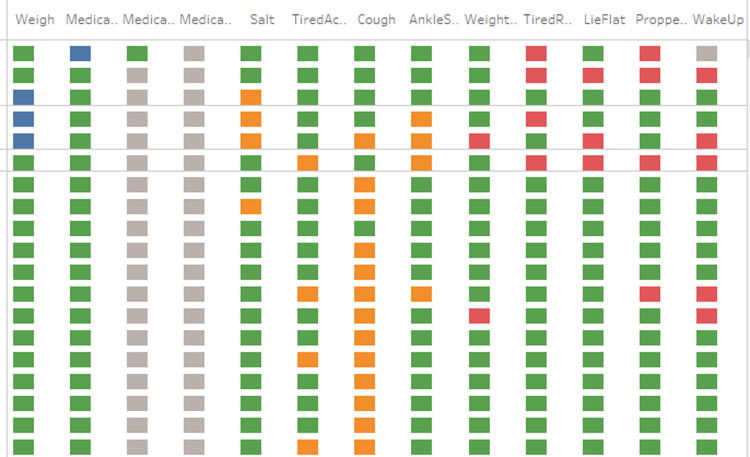
Snapshot from case manager dashboard for an example patient. Each row corresponds to one day. Questions that are greyed out are skipped based on logic (if the patient took his/her medication then follow up questions on medication adherence are not needed). Questions: Weight, Medication All, Medication Most, Medication Some, Salt, Tired Activity, Cough, Ankle Swollen, Weight Increase, Tired Rest, Lie Flat, Propped Up, Wake Up.

In summary, when reviewing patient’s responses green indicated that the patient was stable and was adherent to self-care practices, so green flags did not generate an alert. Flags of any other color, however, indicated that the patient was not adherent (blue), reporting mild HF symptoms (orange), or reporting moderate/severe HF symptoms (red). The study nurse monitored the flags daily including weekends and holidays, and a red flag triggered additional email and text alerts. The text alert gave the participant number and specified the red-flag question the patient had responded yes to. The email alert included this information and showed the dashboard with the color-coded responses (Fig 2). In addition, multiple or persistent red flag responses or an increase in moderate/severe HF symptoms (relative to baseline) constituted a patient change of status. Notification of changes in any patient’s status were provided not only to the study nurse and the study coordinator, but also to the PI, the study physician and the patient’s nurse navigator in the HF clinic.

### Study period

The study period for the Alexa+ patients started on the day of technology installation and training at participants’ residence and ended on the date the device was deregistered or after 90 days, whichever came first. For SOC arm, the start date was defined as consent date adjusted by average duration from consent to start date in Alexa+ arm, and the study duration was the average number of days in the Alexa+ population.

### Outcomes

The primary outcome was the effect of Alexa+ on HF hospitalizations. The secondary outcome was emergency department visit, which provides a more complete utilization analysis. We chose to look at utilization as HF patients persistently incur high costs from rehospitalization and emergency visits and we believe Alexa+ use is well positioned to address early symptoms in HF patients, thereby prevent worsening conditions requiring hospitalization and emergency visits. All patient encounters during each patient’s study period were collected from the EHR. HF related encounters were identified by physician using primary ICD diagnosis codes for HF (S2 Table).

Additionally, we were interested in patients’ experience with the technology, the participants completed pre/post-test technology comfort survey, using a 5-point Likert scales for semi-quantitative measures and free text for qualitative measures. The questionnaire included whether they would use a voice-activated personal assistant in the future to help managing their health, and how they felt about their experiences using Alexa for the study.

### Statistical analysis

Demographics and clinical characteristics data were presented as frequency with percentage for categorical variables and mean ± standard deviation or median with first and third quartile (if non-normal distribution) for continuous variables. Missing data was labeled as NA and summarized but excluded from any statistical testing. Shapiro test were used to test for data normality. For categorical variables Fisher exact test and two-sided Fisher exact test were conducted to test for differences in frequencies. For continuous variables, Student T-test was used to test for differenced in means and Wilcoxon rank sum test was used to test for differences in medians for non-normally distributed continuous variables. HF related hospitalizations or emergency visits data were summarized as frequency and were similarly tested for statistical differences. Finally, data visualization was used to plot each patient flags, encounters and response days over the study period, and visual inspection applied to observe overall trends between patient responses, flags and utilization. Data were analyzed both per protocol and according to the intention-to-treat principles. We presented results of all statistical tests, did not adjust for multiple tests. Tests were two-tailed and a p-vale<0.05 was considered statistically significant. All analyses used deidentified datasets and were conducted with R [24] using the Table 1 and ggplot2 packages.

**Table 1:** Baseline patient characteristics.

	Level	Alexa+ PP	Alexa+ ITT	Standard of care	P-value (PP)	P-value (ITT)
n		20	30	30		
Age, years (mean ±SD)		54.45 ±13.02	55.30 ±12.3	52.93 ±12.16	0.676*	0.457*
Sex (%)	Female	7 (35.0)	12 (40.0)	15 (50.0)	0.386	0.604
	Male	13 (65.0)	18 (60.0)	15 (50.0)		
Race (%)	Black	15 (75.0)	22 (73.3)	25 (83.3)	0.359	0.510
	Asian	0 (0.0)	0 (0.0)	1 (3.3)		
	Other	0 (0.0)	1 (3.3)	1 (3.3)		
	White	5 (25.0)	7 (23.3)	3 (10.0)		
Ethnicity (%)	Hispanic	0 (0.0)	0 (0.0)	1 (3.3)	1	1
	Non-Hispanic	20 (100.0)	29 (96.7)	28 (93.3)		
	South American	0 (0.0)	0 (0.0)	1 (3.3)		
	Unknown	0 (0.0)	1 (3.3)	0 (0.0)		
Body mass index, Kg/m^2^ (mean ±SD)		37.11 ±9.41	36.69 ±9.6)	33.57 ±8.07	0.161*	0.177*
Height, cm (mean ±SD)		173.71 ±7.89	173.73 ±10.1	174.63 ±11.19	0.752*	0.745*
Weight, Kg (mean ±SD)		110.49 ±28.80	109.42 ±28.0	103.42 ±30.28	0.414*	0.429*
Marital status (%)	Divorced/Separated	3 (15.0)	5 (16.7)	7 (23.3)	0.439	0.476
	Living together, not married	3 (15.0)	3 (10.0)	1 (3.3)		
	Married	6 (30.0)	11 (36.7)	10 (33.3)		
	Single, never married	6 (30.0)	6 (20.0)	7 (23.3)		
	Widowed	1 (5.0)	1 (3.3)	5 (16.7)		
	NA	1 (5.0)	4 (13.3)	0 (0.0)		
Annual household income (%)	$0–25,0000	7 (35.0)	10 (33.3)	8 (26.7)	0.296	0.480
	$25,001–50,000	7 (35.0)	9 (30.0)	7 (23.3)		
	$50,001–75,000	0 (0.0)	1 (3.3)	4 (13.3)		
	$75,001–100,000	0 (0.0)	1 (3.3)	3 (10.0)		
	More than $100,000	5 (25.0)	5 (16.7)	8 (26.7)		
	NA	1 (5.0)	4 (13.3)	0 (0.0)		
Education (%)	College graduate (Associates or Bachelors)	3 (15.0)	3 (10.0)	8 (26.7)	0.772	0.504
	High school graduate	5 (25.0)	8 (26.7)	5 (16.7)		
	Post-graduate degree (Masters or Doctorate)	2 (10.0)	2 (6.7)	2 (6.7)		
	Some college	7 (35.0)	10 (33.3)	13 (43.3)		
	Some high school	2 (10.0)	3 (10.0)	2 (6.7)		
	NA	1 (5.0)	4 (13.3)	0 (0.0)		
Duration of HF, years (median [IQR])		4.00 [1.25, 6.00]	5.00 [2.00, 11.00]	4.50 [2.00, 9.25]	0.257^	0.761^
Medications for HF (median [IQR])		7.00 [6.50, 10.00]	8.00 [6.25, 11.00]	8.00 [7.00, 10.00]	0.541^	0.993^

Statistical tests: *two-sided T-test or

^two-sided Wilcoxon rank sum test (if non-normal) for continuous variables and two-sided Fisher exact test for categorical variables (all tests excluded NA).

## Results

The Technical setup of the remote patient monitoring system using voice interface technology included the development of the voice interface and a questionnaire, an application that summarized patients’ answers and setting up an alarm infrastructure. Once deployed, we did not encounter any technology related problems, indicating that the approach may be sustainable and scalable. We discuss the patients’ participation below.

### Study populations

Between December 2018 to November 2019, 10 of the 30 Alexa+ participants were withdrawn from the study due to either becoming ineligible after enrollment (issues with Wi-Fi connection (n = 2); being hospitalized (n = 2) or were lost to follow up (n = 6). In our statistical analysis we therefore represent the Alexa+ arm in two ways. We refer to the full arm of 30 patients as the intention to treat (ITT) arm, and the 20 patients that actively participated in the study as the per-protocol (PP) population. The study concluded due to the final protocol defined event being completed. The average study duration in the PP arm was 87 days. No withdrawals occurred from the SOC arm.

Table 1 summarizes the demographic and clinical characteristics of the SOC, the Alexa+ PP and ITT arms. The Alexa+ arms were majority men (Alexa+ PP: 65%, Alexa+ ITT: 60%) while the SOC arm was equally split. Patients were predominantly Black (Alexa+ PP: 75%, Alexa+ ITT 73.3%, SOC 83.3%) and non-Hispanic (Alexa+ PP: 100%, Alexa+ ITT 96.7%, SOC 93.3%). The average age was 50–55 years (Alexa+ PP: 54, Alexa+ ITT: 55, SOC: 53) and the majority of patients had an annual household income below $50,000 (Alexa+ PP: 70%, Alexa+ ITT 63.3%, SOC 50%). While none of these differences were statistically different, numerically, the SOC arm there were younger, more women, and had a higher average income.

### Healthcare utilization

There was 1(5%) emergency visit over the study period in the Alexa+ PP arm, compared to 0 visit for the SOC arm. We did not observe any hospitalizations in the SOC arm, compared to 3 (corresponding to 15% of the patients) in the PP arm. Utilization differences between the PP and SOC arms was statistically significant with p value 0.021 (Table 2).

**Table 2:** Healthcare utilization summary.

	Alexa+ PP	Standard of care	P-value
n	20	30	
HF Encounter (%)			**0.021**
Emergency department visit	1 (5.0)	0 (0.0)	
HF hospitalization	3 (15.0)	0 (0.0)	
None	16 (80.0)	30 (100.0)	

The differences in number of emergency visits and HF hospitalizations. Statistical tests: two-sided Fisher exact test.

### Patient response and technology utilization patterns

**Fig 3:**
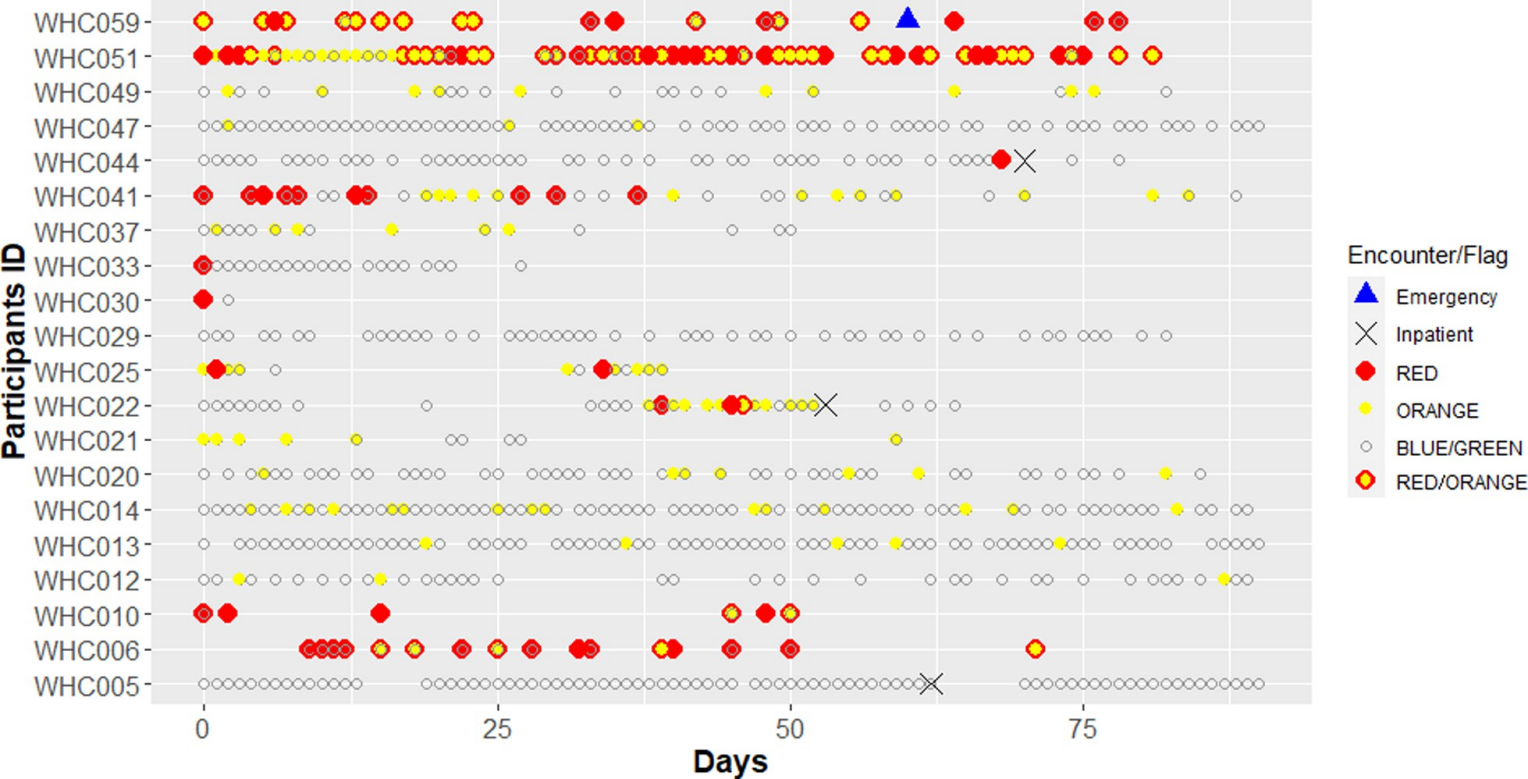
A summary of the flags and health utilization of the Alexa+ PP arm. Grey circles indicate that a patient completed the daily questioner, a red indicates that a red flag was raised, and a yellow dot indicates an orange flag was raised. A yellow dot with red border means both red and orange flags were raised. Utilization is indicated by blue triangles for visits to the emergency room and “x” indicates a HF hospitalization.

All 20 participants in the PP arm interacted with the technology with the mean of using days 34.6 (SD: 23.1) within the study period. Fig 3 summarizes the responses and utilization of the 20 patients. We observed large variability in the clinical course of the HF patients over time. Some patients’ responses never raise a flag, that is the patients are adherent to medication, sodium intake and daily weight, and they did not experience any signs of worsening HF. In contrast, there are patients who consistently self-reported symptoms of HF. We also observed that HF severity (as indicated by the flags) did not directly result in health care utilization.

### Patient experience with Alexa+

The technology comfort survey completion rate was 71% (17/20 patients). Twelve patients felt comfortable/good/liked experience with Alexa+, of whom 4 felt the technology was a good reminder for taking medicine, obtaining daily weight or watching sodium intake, whereas 3 participants felt answering the questionnaire daily was repetitive or redundant;1 participant had difficulty of hearing and responding to device, hence, the participant could not complete without assistance. Moreover, 13 (77%) participants said “Yes” to considering using a voice-activated personal assistant in the future to help to manage their health.

## Discussion

In this clinical trial, we established the feasibility of the use of voice activated technology for remote HF patient monitoring and evaluated its impact on health care utilization (emergency department and HF hospitalizations). The vast majority of HF patients felt comfortable using voice activated technology and indicated willingness to use it in the future for HF disease management. Contrary to our hypothesis, use of voice activated technology was associated with more HF hospitalizations/ED visits. However, these results should be interpreted with caution, owing to the small sample size, imperfect balance of baseline characteristics despite randomization and very low event rate in the study population. The results of this study raise a number of important research questions for future studies.

This study demonstrated feasibility of the use of voice activated technology for remote HF patient monitoring and wide acceptance of the technology among quinquagenarian, predominantly Black and low-income patients with HF. Using a 5-point Likert scales, patients perceived voice activated technology favorably and indicated willingness to engage in voice technology assisted HF remote monitoring. This is very promising, given the unmet need for remote monitoring of HF patients and the advancement in digital technologies. Historically, communities of color and low-income populations have limited access to digital technology. This study demonstrates successful use of that digital technologies and feasibility of bridging the divide gap using the voice assisted technology platform to improve racial disparities in health care.

In this study, use of voice activated technology for remote HF monitoring was associated with more emergency department visits/HF hospitalizations compared with the SOC. It is important to note that, this was a small feasibility clinical trial. While randomization minimizes confounding, it may not completely eliminate residual confounding in small clinical trials. Indeed, we observed numerical differences in some of the baseline characteristics in this study. Actively monitoring patients for signs of worsening health conditions may paradoxically prompt the patient or nurse navigator to utilize health care resources more often. However, this is an unlikely explanation in our study, as 2 of the 3 hospitalizations observed were not preceded by any alarm or a red alarm the day before the HF hospitalization. Conversely, many patients with dynamic alarms did not experience a HF hospitalization or emergency department visit. This underscores the complexity of HF symptomatology and the wide variability in perception and/or reporting of the severity of these symptoms by the patients. The differences observed may be merely by chance and do not reflect a true association between technology utilization and increased health care utilization.

The patient visualization demonstrates the wide range of HF symptom severity experienced by the patients randomized to voice activated technology. This highlights the importance of personalized approach in the design of early warning systems based on patient reported information, by disease severity. Hence, the changes in health status specific to a given patient may be a more reliable indicator of worsening HF. Second, while not all events can be avoided, refinement in the methodology as above, may improve the sensitivity and specificity of the alerts for a worsening HF event that may prompt emergency department visit or a HF hospitalization. An important avenue of future study is the personalization of the study signals, and the quantification of the sensitivity and false alarm rate for the Alexa+ technology for different HF severity. We observe a lack of correlation between HF severity and technology usage that shows that HF severity is not the only driving factor for the technology use. Finally, given the small study population, it is not possible to assess the impact of the technology use based on individual patients’ characteristics, an important question for future research.

The study highlights how currently available voice interface technology can be utilized to build a remote monitoring system. The voice interface technology is not limited to the Alexa+, but can similarly be developed for other voice activated home devices such as the Google Home or Apple’s HomePod. The approach can also be implemented into smartphones through Siri, Cortana, or Google Assistant. The versatility of implementing the developed technology into these devices is promising. Further extensions of the technology can be the incorporation of information from wearable tracking devices (such as an Apple Watch or a Fitbit). With the integration of wearable technology, home monitoring devices can monitor, and remind patients of daily tasks such as daily exercises. The positive impact of technology on communities with large health disparities through improving lifestyle and implementing interventions [13] further encourages additional study into the use of voice technology across platforms and its extensions.

We have conducted a separate study, in part based on the same data, in which we compared predictors of the uptake of two different types of voice-assisted technologies by patients with HF. We found that older patients had higher engagement with telehealth, higher number of HF medications was negatively associated with telehealth use and Black patients had a lower engagement score compared to White patients [25].

When introducing a new technology, inclusivity of different patient populations is important. Comorbidities, cultural background, and economic status are some of the important considerations. Pronunciation/enunciation is an important factor when considering the accessibility of the technology. Adaptation of the technology into a variety of languages will help provide inclusivity for a non-native English speaker and minimize errors in data collection and therefore increase the value that the technology can bring to the patients. Another important group are the deaf and hard of hearing who would not be able to utilize these devices. Patients with cognitive impairments may also not be able to use these devices. Lastly, digital literacy is highly variable among patients and additional training on digital literacy and device use may be needed. Health disparities are often correlated with economic status [26, 27]. Many families with low income may not be able to afford or access internet service or an at-home digital device that can aid in monitoring their health. Development of programs to achieve digital equity by expanding access and ensuring affordability of high-speed internet and digital devices and investing in digital literacy and support programs.

In the wake of the COVID-19 pandemic, millions of medical staff have begun to voice the impact of the catastrophic pandemic on their mental, emotional, and physical health [28, 29]. The utilization of remote monitoring devices may decrease the strain felt on medical staff, particularly on nurses, medical assistants, and patient techs. Rather than have a medical staffer arrive at 60 patients’ homes daily, it is much easier to utilize at home devices to gather data on 60 patients daily. Instead of daily in-person check-ins, a medical staffer can have a weekly check in with the 60 patients yet still collect adequate data on their wellbeing. The technology developed can be adopted for any patient population that may need daily monitoring. This platform could also decrease the need for in-person interactions while keeping the close provider-patient connection, which would improve safe patient care, in the setting of a pandemic.

## Conclusions

The future of healthcare is personalized and individualized. This study is a step towards patient empowerment and a personalized approach to HF care. HF is a chronic and serious disease that requires active self-care, with which patients often struggle. Utilizing voice activated technology that is currently publicly available is an effective way to empower patients and support daily monitoring of HF patients. Alerting patients, and providers of worsening conditions, aids in preventing further complications. Further research is needed to fully tailor this technology to different levels of HF severity, to maximize its potential as an aid in self-monitoring for HF and different chronic illnesses more generally.

## Supporting information

S1 Checklist CONSORT 2010 checklist of information to include when reporting a randomised trial*. (DOC)

S1 Table Full HF care survey script of Alexa. (DOCX)

S2 Table Full list of HF related ICD-10 codes. (DOCX)

S1 Protocol Study protocol. (PDF)
