# Supplementary material for: Voice activated remote monitoring technology for heart failure patients: Study design, feasibility and observations from a pilot randomized control trial
Source: PLoS One. 2022 May 6;17(5):e0267794. doi: 10.1371/journal.pone.0267794 (PMC9075666; doi:10.1371/journal.pone.0267794)
Supplement: S1 Table — (DOCX) [file pone.0267794.s002.docx]

**S1 Table. Full HF care survey script of Alexa+**

| **HF Care Survey Script** |
| --- |
| **First-Time Intro:** |
| Hello and Welcome!  I am PAI/Alexa, your Digital Health Assistant. |
| I will be asking you a series of questions, to which you need to answer a simple “YES” or “NO”. |
| Let’s get started with your heart health survey! |
| **Everyday Intro:** |
| * Welcome back.  Let’s see how you are doing today. [weigh q1] |
| * Hi. Let's get started with your heart health survey. [weigh q1] |
| * Hello.  Let’s complete your heart health survey for today. [weigh q1] |
| * Hello again. Let’s get started with your heart health survey. [weigh q1] |
| * Good to talk with you again. Let’s go through your heart health survey for today. [weigh q1] |
| **Compliance:** |
| 1.       Did you weigh yourself today? (Yes = next question; No = prompt to weigh) |
| If answer is YES, |
| * Great, then. [med q2] |
| * Excellent. And [med q2] |
| * Okay! [med q2] |
| * Good. And [med q2] |
| * Wonderful. Now, [med q2] |
| If answer is NO, |
| * Ok - Please weigh yourself after you complete this survey [med q2] |
| * Got it. Please weigh yourself every after you finish this survey. [med q2] |
| * Ok, remember to weigh yourself after you finish this survey. And [med q2] |
| * It's important to weigh yourself every day. Please weigh yourself after you complete this survey. Now, [med q2] |
| 2.a      Did you take all your heart failure medications as prescribed since the last survey? (Yes = next question; No = Ask if took most) |
| If answer is YES, |
| * Wonderful, [salt q3] |
| * Good to hear. [salt q3] |
| * Great. [salt q3] |
| * Good! [salt q3] |
| * Okay, [salt q3] |
| If answer is NO, |
| 2.b       Did you take most of your heart failure medications as prescribed since the last survey? (Yes = next question; No = Ask if took some) |
| If answer is YES, |
| * Okay. Please be sure to always take all your medications as prescribed. [salt q3] |
| * Okay, but it’s important to take all your medications as prescribed every day. [salt q3] |
| * Good to hear but please be sure to take all your medications exactly as prescribed every day. [salt q3] |
| If answer is NO, |
| 2.c       Did you take some of your heart failure medications as prescribed since the last survey? (Yes = next question; No = Remind them to take) |
| If answer is YES, |
| * Okay. Please be sure to always take all your medications as prescribed. [salt q3] |
| * Okay, but it’s important to take all your medications as prescribed every day. [salt q3] |
| * Fine but please be sure to take all your medications exactly as prescribed every day. [salt q3] |
| If answer is NO, |
| * Please be sure to take your medications right after we finish this survey. [salt q3] |
| * Please be sure to take your medications after you complete the rest of the survey. It's important to take your medications every day. [salt q3] |
| * It is very important you take your medications exactly as prescribed.  Please do it after we are done with your heart health survey [salt q3] |
| * Following your medication prescription is very important - please take your medications after you finish your heart health survey. [salt q3] |
| 3.       Did you eat any high salt foods since the last survey? (Yes = reminder that should take no added salt and be restricted to 3 gram of salt/day; No= next question) |
| If answer is NO, |
| * Keep it up, now [tired activity q4] |
| * Good. And [tired activity q4] |
| * Okay, [tired activity q4] |
| * Great to hear! [tired activity q4] |
| * Perfect.[tired activity q4] |
| If answer is YES, |
| * Remember to keep your salt under 3 grams a day, and don't add additional salt to your food. [tired activity q4] |
| * Please keep your salt intake under 3 grams a day, it’s important for your health. [tired activity q4] |
| * Remember, no more than 3 grams of salt a day, and don't add extra to your food. [tired activity q4] |
| * Your doctor recommends that you keep your salt under 3 grams a day, and refrain from adding extra salt at the table. [tired activity q4] |
| **HF Symptoms** |
| **Mild:** |
| 4.       Do you feel tired or short of breath with regular daily activities such as eating or taking a |
| shower? (Yes =orange; No=next question) |
| If answer is NO, |
| * Positive Interjection, [cough q5] |
| If answer is YES, |
| * Negative Interjection, [cough q5] |
| 5.       Do you have cough or wheezing? (Yes = orange; No=next question) |
| If answer is NO, |
| * Positive Interjection, [ankles q6] |
| If answer is YES, |
| * Negative interjection [ankles q6] |
| 6.       Are your ankles swollen? (Yes = orange; No = next question) |
| If answer is NO, |
| * Positive interjection [weight increase q7] |
| If answer is YES, |
| * Negative interjection. [weight increase q7] |
| **HF Symptoms** |
| **Moderate to severe:** |
| 7. Did your weight increase by 3 pounds or more in 1 day? (Yes = Red flag) |
| If answer is NO, |
| * Positive interjection. [tired rest q8] |
| If answer is YES, |
| * Negative interjection [tired rest q8] |
| 8.       Do you feel tired or short of breath at rest (for example when sitting)? (Yes = Red flag) |
| If answer is NO, |
| * Positive interjection. [short flat q9] |
| If answer is YES, |
| * Negative interjection [short flat q9] |
| 9.       Do you feel short of breath when you lie flat in bed? (Yes = Red flag) |
| If answer is NO, |
| * Positive interjection [pillow q10] |
| If answer is YES, |
| * Negative interjection [pillow q10] |
| 10.       Do you need to be propped up with pillows in order to be able to sleep without shortness of breath? (Yes = Red flag) |
| * Okay, we’re almost done. [situp q11] |
| * Almost there. [situp q10] |
| * Final question. [situp q10] |
| * Right. Last question, [situp q11] |
| * And lastly, [situp q10] |
| 11.       Do you wake up from sleep in the middle of the night with shortness of breath and need to sit up to feel less short of breath? (Yes = Red flag; No= end of Questions) |
| * Thank you for completing your heart health survey. |
| * You are all done with your heart health survey. |
| * You have completed your heart health survey for today. |
| If “RED / MODERATE TO SEVERE SYMPTOMS”, |
| If “WEIGHED SELF” & “TOOK MEDS” & “NOT MUCH SALT” |
| // Do nothing |
| Else If “WEIGHED SELF” & “TOOK MEDS” |
| * Please make sure to keep your salt intake under 3gms” |
| * Remember it is very important to comply with the heart failure diet.” |
| Else If “WEIGHED SELF” & “NOT MUCH SALT” |
| * Please remember to take your medications as prescribed after the survey.” |
| * It is very important that you take your medications as prescribed after we are done.” |
| Else If “TOOK MEDS” & “NOT MUCH SALT” |
| * Please remember to weigh yourself after we are done.” |
| * It is important you weigh yourself after you finish the survey. |
| Else If “WEIGHED SELF” |
| * Please remember to take your medications when we are done as prescribed, and to keep your salt intake under 3gms. |
| Else If “TOOK MEDS” |
| * Please remember to weigh yourself and record your weight when we are done, and to comply with the heart failure diet. |
| Else |
| * Please remember to weigh yourself when we are done, and to take your medications as prescribed. |
| If “WEIGHED SELF” & “TOOK MEDS” & “NOT MUCH SALT” |
| * You did well, today, weighing yourself, taking your medications, and complying with heart failure diet” |
| *Well done, today, you weighed yourself, took your medications, and complied with the heart failure diet” |
| * Way to go, weighing yourself, taking your medications, and keeping your salt intake under 3gms, today.” |
| Else If “WEIGHED SELF” & “TOOK MEDS” |
| * Good job, today. You just need to keep your salt intake under 3gms” |
| * You did well today. You just need to comply with the heart failure diet.” |
| Else If “WEIGHED SELF” & “NOT MUCH SALT” |
| * Good job, today. You just need to take your medications as prescribed” |
| * You did well, today. You just need to take your medications as prescribed to make it perfect” |
| Else If “TOOK MEDS” & “NOT MUCH SALT” |
| * Good job, today. You just need to weigh yourself after we are done.” |
| * You did well, today. You just need to weigh yourself after we are done to make it perfect” |
| Else If “WEIGHED SELF” |
| * Please remember to take your medications when we are done, as prescribed, and to keep your salt intake under 3gms. |
| Else If “TOOK MEDS” |
| * Please remember to weigh yourself and record your weight when we are done, and to comply with the heart failure diet. |
| Else |
| * Please remember to weigh yourself and record your weight when we are done, and to take your medications as prescribed. |
| IF “RED / MODERATE TO SEVERE SYMPTOMS” |
| * “X”, from what you told me, you are experiencing the following symptoms: |
| If “WEIGHT INCREASED” |
| * Your weight has increased more than 3 pounds in the last day. |
| If “TIRED REST” |
| * you feel tired or short of breath when you rest. |
| If “SHORT FLAT” |
| * you feel short of breath when you lie flat in bed. |
| If “PILLOW” |
| * you need to be propped up with pillows in order to be able to sleep without shortness of breath. |
| If “SITUP” |
| * you wake up from sleep in the middle of the night with shortness of breath and need to sit up to feel less short of breath. |
| This could be a sign of worsening heart failure. Please notify your heart failure nurse navigator. If this is a medical emergency, please call 911. |
| Finally, please remember that I am a virtual assistant and not a health care professional. Please use your judgment and seek medical attention if you are not feeling well or have concerns about your health. |
| * Have a good day and talk tomorrow! |
| * Good bye! |
| * Take care! |
| * See you tomorrow.  Good bye! |
| * Hope you have a good day. Bye! |
| **Positive Interjections (Randomly chosen):** |
| 1. * Got it. |
| 2. * Okay. |
| 3. * All right! |
| 4. * Excellent. |
| 5. * That's good, |
| 6. * Fine. |
| 7. * Right. |
| 8. * Nice. |
| 9. * Very Good. |
| 10. * Oh good. |
| 11. * Great. |
| 12. 'Good' |
| 13. ‘That’s great’ |
| 14. ‘Wonderful’ |
| **Negative Interjections (Randomly chosen):** |
| 1. * Okay, |
| 2. * Right, |
| 3. * Okay, I see. |
| 4. * Oh, I see. |
| 5. * Got it. |
| 6. * Alright |
| 7. * I see. |
| 8. ‘Sorry to hear that’ |
| 9. All right! |
