# Supplementary material for: Voice activated remote monitoring technology for heart failure patients: Study design, feasibility and observations from a pilot randomized control trial
Source: PLoS One. 2022 May 6;17(5):e0267794. doi: 10.1371/journal.pone.0267794 (PMC9075666; doi:10.1371/journal.pone.0267794)
Supplement: S2 Table — (DOCX) [file pone.0267794.s003.docx]

**S2 Table. Full list of HF related ICD-10 codes**

Diagnosis codes of patient encounters were reviewed for study participants. This list represents heart failure specific visits and heart failure related visits, including comorbidities associated with heart failure.

The following ICD-10 codes were identified by physician as HF related diagnoses. The encounters with any of the codes in primary or secondary diagnoses were counted as HF related utilization for the patients.

| **ICD-10 CODE** | **ICD-10 CODE DESCRIPTION** |
| --- | --- |
| I50 | Heart failure |
| I50.1 | Left ventricular failure, unspecified |
| I50.2 | Systolic (congestive) heart failure |
| I50.20 | Unspecified systolic (congestive) heart failure |
| I50.21 | Acute systolic (congestive) heart failure |
| I50.22 | Chronic systolic (congestive) heart failure |
| I50.23 | Acute on chronic systolic (congestive) heart failure |
| I50.3 | Diastolic (congestive) heart failure |
| I50.30 | Unspecified diastolic (congestive) heart failure |
| I50.31 | Acute diastolic (congestive) heart failure |
| I50.32 | Chronic diastolic (congestive) heart failure |
| I50.33 | Acute on chronic diastolic (congestive) heart failure |
| I50.4 | Combined systolic (congestive) and diastolic (congestive) heart failure |
| I50.40 | Unspecified combined systolic (congestive) and diastolic (congestive) heart failure |
| I50.41 | Acute combined systolic (congestive) and diastolic (congestive) heart failure |
| I50.42 | Chronic combined systolic (congestive) and diastolic (congestive) heart failure |
| I50.43 | Acute on chronic combined systolic (congestive) and diastolic (congestive) heart failure |
| I50.8 | Other heart failure |
| I50.81 | Right heart failure |
| I50.810 | unspecified |
| I50.811 | Acute right heart failure |
| I50.812 | Chronic right heart failure |
| I50.813 | Acute on chronic right heart failure |
| I50.814 | due to left heart failure |
| I50.82 | Biventricular heart failure |
| I50.83 | High output heart failure |
| I50.84 | End stage heart failure |
| I50.89 | Other heart failure |
| I50.9 | Heart failure, unspecified |
| I11.0 | heart failure due to hypertension |
| I13.0 | heart failure due to hypertension with chronic kidney disease |
| I13.2 | Hypertensive heart and chronic kidney disease with heart failure and with stage chronic kidney disease, or end stage renal disease |
| I97.130 | heart failure following cardiac surgery |
| I97.131 | heart failure following other surgery |
| I09.81 | rheumatic heart failure |
