# Supplementary material for: Voice activated remote monitoring technology for heart failure patients: Study design, feasibility and observations from a pilot randomized control trial
Source: PLoS One. 2022 May 6;17(5):e0267794. doi: 10.1371/journal.pone.0267794 (PMC9075666; doi:10.1371/journal.pone.0267794)
Supplement: S1 Protocol — (PDF) [file pone.0267794.s004.pdf]

SPECIFIC AIMS

Voice enabled, internet connected devices are poised to have profound impacts on the quality of life for in-home, independent, and assisted-living patients by vastly increasing the connectivity of patients with family as well as healthcare providers. Amazon Echo Dot (Alexa) is a voice command intelligent virtual personal assistant that functions through interfacing with smart devices and platforms to provide interactive actions and assistances with various tasks. Integrating data from virtual voice activated assistants (Amazon Echo Dot with Alexa voice technology), with the rich medical history available in our suite of Electronic Health Records (EHR) allows us to create a truly customized, interactive, and automated personal healthcare assistant. We propose to validate this technology in patients with the MedStar health system who have chronic heart failure (HF). We will recruit subjects from MedStar heart failure clinic and the MedStar's Washington Hospital Center inpatient heart failure service. MedStar Health is the largest nonprofit health-care delivery system in the Washington, DC/Baltimore area with more than 4 million outpatient visits and 200, 000 inpatient visits annually.

Following a 1:1 randomization, half of the study patients will receive an Amazon Echo Dot (Alexa) enhanced with a HF Care custom Alexa Skill that is patient-specific, which will be referred to in this proposal as Alexa+. Data on patient interactions with the device will be followed for three months. The control groups will receive standard of care (SOC) HF management. Both study and control groups will receive regular follow up from the HF nurse and data from all hospital or clinic visits will be captured from the EHR. Data generated from Alexa and clinical data from the EHR will be integrated to create the analytical platform for our data and methods validation. We have developed the following aims:

Specific aim 1:

To design and test a customized and interactive chronic heart failure disease specific functionality (skill kit) within a voice activated technology (Amazon's Echo Dot) as a tool for management of patients at home.

Hypothesis:

Designing a customized and interactive chronic heart failure skill kit within a voice activated technology (Amazon's Echo Dot) for management of patients at home is feasible and is accepted by technology savvy as well technology naïve patients.

Specific Aim 2:

To identify the effect of a voice activated technology (Amazon's Echo Dot) in reduction of heart failure hospitalizations, improvement of medication adherence, improvement in health related quality of life and increase in comfort with voice activated technologies.

Hypothesis:

Utilization of Amazon's Echo Dot as a virtual assistant for patients with chronic heart failure will result in reduction of heart failure hospitalizations, improvement of medication adherence, improvement in health related quality of life and increase in comfort with voice activated technologies.

Research Strategy

1.SIGNIFICANCE

Social media and digital engagement between patients and providers are becoming more prevalent for care coordination and management in healthcare. Voice activated technology (e.g. Amazon's Echo Dot) can be programmed with additional enhancements over their baseline/native functions. These technologies have the potential to empower patients to better manage their own health by setting medication or wellness reminders, offering real time access to health care information, and even enabling patients with similar diagnoses interact with one another. However, like all technological interventions, not everyone is amendable to these systems nor will all patients benefit equally from them. To tease apart these issues we propose to test the Echo Dot for increasing positive healthcare outcomes in a chronic HF population.

Technology can lead to significant long-term cost savings and can decrease provider burden, yet there is much more that can be done to manage the needs of patients. Although elderly adults who are poor, uneducated, or disabled tend to struggle to adopt new technologies[1], many seniors are eager to try. According to an Accenture survey [2], 67% of tech-savvy seniors would like to access health services from home. They show an interest in proactively managing their health: ~25% said they regularly accessed their EHR Patient Portal, and over 60% would wear a health monitoring device and engage with online health-related communities. Findings from a recent Pew Research Center [3] survey show that seniors are increasingly using the internet to communicate with family and friends, shop for goods and services, and access healthcare information. College graduates under 75 years old who earn more than $75,000/year are the most comfortable with technology and the most eager to embrace it[3]. Tech-resistant seniors reported a lack of confidence with technology and were also likely to report medical conditions such as poor eyesight or other disabilities as barriers to using technology[3]. But despite these heterogeneous comfort levels, nearly all seniors expressed an interest in leveraging technology to meet their individual medical needs. A technological divide exists between seniors, but better patient engagement technology can bridge this divide[4].HF is the quintessential complex disease of the elderly population, where care is delivered at different settings. HF is prevalent in 6.5 million adult Americans[4]. The incidence of HF rises by nearly two-folds in men with each 10-year age increase from age 65-85 years and by three-folds in women between ages 65 year-74 years and 75-84 years[4]. HF is one of the most common discharges diagnoses and is the most frequent cause of 30-day hospital readmission among the Medicare beneficiaries accounting for a huge societal burden[5]. Optimization of health care delivery in different settings with the goal of improving quality of life and reduction of HF hospitalizations is an unmet need.

Adopting a voice-activated assistant in managing care-coordination would provide long-term savings through reducing hospitalizations and eliminating effort duplication[6]. Better care coordination would also decrease provider burden by saving time and precious hospital resources. For instance, a personalized voice activated assistant would eliminate the need for nurses to remind patients of their medications on a daily basis. It

could also lead to higher rates of adherence, thereby benefiting hospital systems in the long-run.

2. INNOVATION

Alexa is Amazon’s voice platform and the brain behind millions of devices like the Amazon Echo. In addition to its well-publicized features (voice interaction, music playback, creating to-do lists, setting alarms, streaming podcasts, playing audiobooks, and providing real time information, such as news, weather, and traffic) Alexa also provides developers the opportunity to add to and augment Alexa’s capabilities (known as ‘skills’). There are now more than 13,000 skills from companies like Starbucks, Uber, and Capital One, as well as innovative designers and developers. We will use an Alexa-powered device called ‘Echo Dot’. Echo Dot is a fully functional miniaturized version of the original ‘Echo’ device, and retails at $50/unit. Echo and Echo Dot are always connected to Amazon's Alexa cloud network. As one of those Alexa appliances is used, it learns user- preferences, speech patterns, and vocabulary, to better deliver on what it is being asked. In this proposal we expect to be able to see and measure improvements in patient-provider interactions, and empower patients to play a more active role in their daily health. Because Alexa is voice activated, and not reliant on a touchscreen or keyboard, we anticipate that both tech-savvy and tech- naïve patients will benefit from this technology. In our study an augmented version of Alexa running on the Echo Dot, called Alexa+ will ask patients a series of questions. Based on the answers to these questions, it will either contact a study coordinator or will ask patients to take a specific action such as weighing themselves. If a patient responds to Alexa+ that she or he has shortness of breath, or swelling of the ankles, then Alexa+ will contact the study coordinator who will bring it to the attention of the physician treating that patient. Data from this simple intervention will be merged with data extracted from the patient’s health record, and analyzed for clinical variables and demographics that are most predictive for success in use of these technologies. These potential technologies and systems can boost the amount of healthcare data we can gather on a patient[7,8], instead of relying solely on hospital and/and outpatient encounters, which can be much more sporadic. Increasing the amount of data we have for a particular patient will allow us to use machine learning techniques to customize certain components of a patient care plan in close to real time[9]. This has particular application in chronic heart failure patients who may have co-morbidities which often involves managing care transitions between specialists and across different settings. Voice-activated assistants would provide long-term cost savings by (1) decreasing provider burden by saving time and limited hospital resources, e.g. eliminating the reliance upon nurses to remind patients of their medications on a daily basis; (2) reduce hospitalizations by increasing medication adherence and preventing complications by identifying and attending to early warnings of adverse events.

This is the first project in which this type of data will be integrated with MedStar’s patient health records. MedStar Health Research Institute is a member in the Georgetown-Howard Universities Center for Clinical and Translational Science (GHUCCTS). GHUCCTS was funded to accelerate the translation of discoveries into improved patient care and public health. This partnership of Georgetown University, Howard University, MedStar Health Research Institute, the Washington DC Veterans Administration

medical Center and Oak Ridge National Laboratory has taken notable steps to meet this goal since 2010. The strategic goals of the consortium is to improve the infrastructure that supports high quality interdisciplinary clinical and translational research; to continue to develop GHUCCTS as a model of inter-institutional collaboration; to leverage our location, expertise to design and implement research that will have a high impact on underserved populations with health disparities, including minorities, people with disabilities, and elderly population; to design and disseminate vibrant and innovation educational programs; and finally, to draw from and contribute to the collective efforts of the national CTSA consortium to advance the strategic goals of GHUCCTS, the National Center for Advancing Translational Science, and the CTSA national consortium.

Figure 1: Study flow and timeline

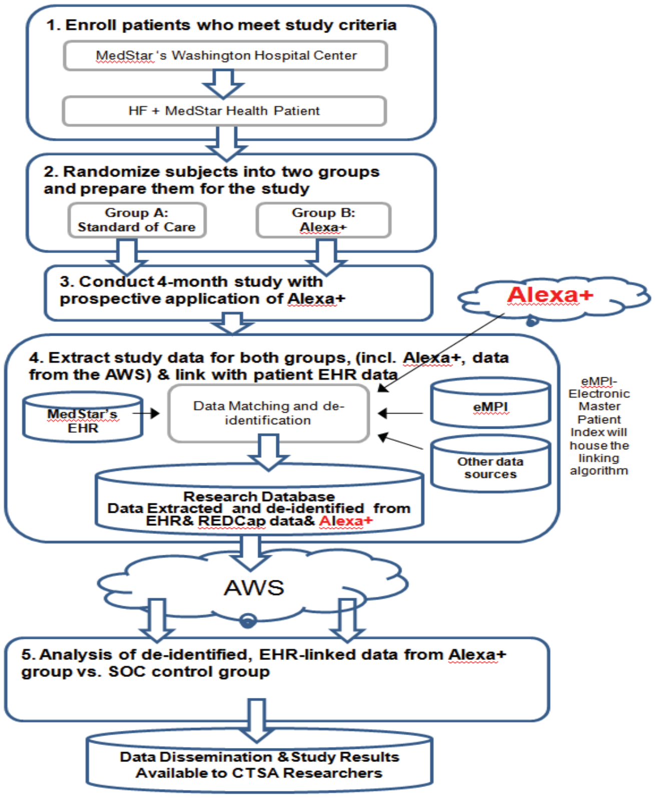

Our platform will be a shared informatics collaboration space linked with the informatics core throughout the CTSA consortium. We wish to ensure that the platform is broadly usable by non-technologists with a particular emphasis on training the new generation of healthcare providers who manage and care for complex patients. If necessary our data will be made interoperable via mapping to common data models to merge with other data for mining and modeling.

3. APPROACH

Specific aim 1:

To design and test a customized and interactive chronic heart failure disease specific functionality (skill kit) within a voice activated technology (Amazon's Echo Dot) as a tool for management of patients at home.

We will leverage the patients' electronic medical record and extract relevant clinical data to assist with the design of the tool.

Alexa Skill Kit (ASK) allows developers to build new 'skills' for Alexa and thus develop customized applications. ASK is a collection of self-service APIs, tools, documentation, and code samples that enable developers to add skills to Alexa. Users access these new skills by asking Alexa a question using the newly created command words that trigger the response. The ASK development environment is free to use and free cloud-hosting services are offered on Amazon Web Services (AWS). We plan to use ASK to develop the Alexa Skill that asks patients a set of customized HF Care questions, the HF Care Alexa Skill. For the purpose of this study, the HF Care Alexa Skill running on

Amazon Echo Dot units is being referred to as Alexa+. **Alexa+ Study:** We will recruit, consent, and enroll a total of 50 patients from the heart failure clinic at the MedStar's Washington Hospital Center, 25 patients will be randomized to the Alexa+ group and 25 patients to the SOC group. Study workflow is shown in Figure 1. Patients randomized to the Alexa+ study group will be given an Amazon account and equipped with an Echo Dot configured to access the HF Care Alexa Skill. Note, each user will have an Amazon account allowing them to use their Echo Dot set to access HF Care Alexa Skill. We will provide training for the HF study group on how to use their Alexa+ Echo Dot sets including Alexa Voice Training. Voice Training consists of a session of 25 phrase repetitions that allows Alexa to improve its voice recognition capabilities for the target user.

Participants will be provided the Alexa+ units for 3 months, and weekly check-in by phone from the study coordinator will help answer any questions and provide additional training to the participants on how to invoke and communicate with it as outlined for the Alexa+ group. For the purpose of this study, we selected a series of simple yes/no questions (HF Care Alexa Skill) resulting in either the next question/action in the decision tree, or prompting Alexa+ to notify the study coordinator as follows:

-Do you feel short of breath when you lie flat in bed? (Yes = contact study coordinator; No=next question)

-Do you need to be propped up with pillows in order to be able to sleep without shortness of breath? (Yes = contact study coordinator; No=next question)

-Do you wake up from sleep in the middle of the night with shortness of breath and need to sit up to feel less short of breath? (Yes = contact study coordinator; No=next question)

- Do you feel fatigued or short of breath at rest (when doing nothing)? (Yes = contact study coordinator; No=next question)

-Do you feel fatigued or short of breath with regular daily activities such as taking a shower, dressing, eating, cooking, walking a short distance on the level or simple cleaning? (Yes = contact study coordinator; No=next question)

-Do you have cough or wheezing? (Yes = contact study coordinator; No=next question)

-Are your ankles swollen? (Yes = contact coordinator; No = next question)

-Did you weigh yourself? (Yes = next question; No = prompt to weigh)

-Did you take your medications today? (Yes = next question; No = contact coordinator)

-Did you eat any high salt food today? (Yes = contact study coordinator; No=end of questions)

The overarching goal for this work is to use data generated from this proof of concept to create the HF decision tree that will help stratify HF patients into the HF zones as shown in Figure 2.

This will help patients engage in taking better self-care on a daily basis and reduce the time required from nurses and physicians to conduct daily calls to alert and/or remind patients to answer simple questions. Alexa+ results will be accumulated and stored in AWS and available for retrieval at anytime. At the end of the 3 months, we will extract and organize the Alexa+ survey results for the HF Group study participants. These data will be combined with medical data abstracted from the MedStar EHR (see below), and the results of the post-test survey. Data will be analyzed as described in Aim 2.

<u>EHR & Survey Data Management:</u> Drs. Shara and Brooks are the MHRI gate-keepers to an extensive array of health IT tools. MedStar Health operates over 185 separate information system, of which a little over 100 can be considered clinical. The majority of those that capture patient information transfer their data to one or more patient data repositories, depending on the nature of the patient and the data. *Cerner MedConnect* is the enterprise suite of clinical applications which collectively provide the foundation of the EHR for our patients. Data harvesting occurs via a clinical data repository and viewer called Amalga. Where reasonable we will impute missing values and visualize using Tableau software to ensure no deviation from biological possibility, and to confirm trends remain intact, otherwise data is flagged as missing. Although missing data pose problems, we expect our research to gather enough patient data that missingness does not skew our output from Aim 2. Study groups will be asked to rate their comfort with using technology by a pre- and post-test technology survey, which will be administered by study coordinators using the well validated REDCap clinical data management system. Dr. Brooks' staff administer MedStar REDCap instance. REDCap is a secure, web-accessible, user-friendly clinical data collection interface communicating with an encrypted MySQL database. REDCap offers a mobile app for offline data collection, and all users will be trained to use secure internet connections only (i.e. VPN; no public Wi-Fi; use Ethernet when possible). Study coordinators will administer the surveys and questionnaires after the patient is randomized to either the study or control arm. Our pre/post-test technology comfort survey uses 5-point Likert scales for quantitative measures and free text for qualitative measures. Free text will be exposed to natural language programming to generate additional structured variables. We will also use text-analysis (Carrot2 on Apache Lucene indexes etc.) to help quantify mood and contextual cues. These data will augment the basic clinical variables and be useful for identifying ideal patients for these interventions. A slew of validated questionnaires are readily available in REDCap, we will administer the following validated questionnaires (SF-36: quality of life, Hospital Anxiety and Depression Scale (HADS) and Health-related quality of life (HRQOL) to assess the psychosocial aspects of the subjects participating in this study. At the end of the study EHR, Alexa+ and REDCap data will be merged and hosted in a secure, HIPAA compliant MySQL database on a private virtual machine within the MedStar IS managed cluster and mapped to RStudio for analysis (Aim 2).

Specific Aim 2:

To identify the effect of a voice activated technology (Amazon's Echo Dot) in reduction of HF hospitalizations, improvement of medication adherence, improvement in health related quality of life and increase in comfort with voice activated technologies.

Statistics will be computed using a user-made function to summarize data bivariately. Our customized package employs the doBy package in R to compute stratified summary statistics, and uses the R2wd and RDCOMClient packages to export those results into a MS Word document. Within the Alexa+ group, we will ascertain whether Alexa+ has been adopted and if the questions prompted reliable adherence. Ideally, each patient in the Alexa+ group should have four positive responses to the four prompts. Equally we will examine patients with negative responses to the prompts. However, we expect to see some degree of missingness in the responses and this

missingness may be correlated with the passage of time. Increasing missingness over time indicates a weariness effect, while decreasing missingness overtime indicates a degree of adoption. This missingness will be analyzed using time series analysis functions such as stl() and arima() found in the R base package. The rate of missingness will be plotted using the ggplot() function within the ggplot2 package of R. Pattern in adherence to the prompts will be analyzed, again using time series. We will also use a logistic model to determine if the odds of adherence are higher at the end of the time period compared to the beginning of the time period. Comparison between control and study groups will be performed using the survival package in R as well as the base functions. After the Alexa+ information is merged with the EHR data, readmission will be assessed using a survival regression adjusted so as to model for multiple readmissions if necessary. Data will be restructured using the Reshape2 package so that survival to each event will be considered an independent entry. Medication adherence will be modeled using a similar survival regression, but instead modeling when a new prescription is filled. To assess the acceptability of Alexa within the group, a linear model will be fit on the scores of the post-survey test (see Aim 1). The regression will control for age, income, co-morbidities, and compensate for degree of non-missingness and adherence to Alexa+ prompts. We predict that study participants who use the technology a lot are more likely to be satisfied with the experience and will find Alexa an acceptable tool for their needs monitoring.

Figure 2: Heart Failure Zones

EVERY DAY	**EVERY DAY:**<br>• Weigh yourself in the morning before breakfast, write it down and compare to yesterday's weight.<br>• Take your medicine as prescribed.<br>• Check for swelling in your feet, ankles, legs and stomach.<br>• Eat low salt food.<br>• Balance activity and rest periods.<br>**Which Heart Failure Zone are you today? GREEN, YELLOW or RED?**
GREEN ZONE	**ALL CLEAR – This zone is your goal**<br>Your symptoms are under control. You have:<br>• No shortness of breath.<br>• No weight gain more than 2 pounds (it may change 1 or 2 pounds some days).<br>• No swelling of your feet, ankles, legs or stomach.<br>• No chest pain.
YELLOW ZONE	**CAUTION – This zone is a warning**<br>Call your doctor's office if:<br>• You have a weight gain of 3 pounds in 1 day *or* a weight gain of 5 pounds or more in 1 week.<br>• More shortness of breath.<br>• More swelling of your feet, ankles, legs, or stomach.<br>• Feeling more tired. No energy.<br>• Dry hacky cough.<br>• Dizziness.<br>• Feeling uneasy, you know something is not right.<br>• It is harder for you to breathe when lying down. You are needing to sleep sitting up in a chair.
RED ZONE	**EMERGENCY**<br>**Go to the emergency room or call 911 if you have any of the following:**<br>• Struggling to breathe. Unrelieved shortness of breath while sitting still.<br>• Have chest pain.<br>• Have confusion or can't think clearly.

EXPECTED OUTCOMES & DELIVERABLES: In addition to demonstrating that augmented voice-responsive devices such as Alexa+ are feasible and well accepted by patients with chronic illnesses, we anticipate that this technology will enhance patient's quality of life and improve health outcomes. We further expect to be able to identify patients who are most receptive of, and, responsive to, these forms of technological interventions. These results will be broadly useful throughout the community and should readily translate to any other large, urban setting. Although our efforts are here initially focused on HF patients, we expect to be able to extrapolate from demographic factors and health data to other chronic conditions. We will share with our colleagues a suite of tools and data allowing others to build upon our work. Finally, we are hopeful that this work will lead us further towards our end of goal of autonomous mobile Health support for chronically ill patients, their families, and their providers.

References:

1. Khosravi, P. and Ghapanchi, A.H. (2016). Investigating the effectiveness of technologies applied to assist seniors: A systematic literature review. *Int J Med Inform.* 85 (1): 17-26.
2. Accenture, March (2015). “Tech Savvy Seniors want Online Options to Access Care from Home”. Available at https://newsroom.accenture.com/industries/health-public-service/tech-savvy-seniors-want-online-options-to-access-care-from-home-accenture-survey-shows.htm
3. Pew Research Center, April (2014), “Older Adults and Technology Use”. Available at: http://www.pewinternet.org/2014/04/03/older-adults-and-technology-use/
4. Benjamin EJ, Blaha MJ, Chiuve SE, et al. (2017). Heart Disease and Stroke Statistics-2017 Update: A Report From the American Heart Association; American Heart Association Statistics Committee and Stroke Statistics Subcommittee. Circulation Mar 7;135(10):e146-e603. PMID: 28122885.
5. Jencks SF, Williams MV, Coleman EA. (2009). Rehospitalizations among Patients in the Medicare Fee-for-Service Program. N Engl J Med 2009; 360:1418-28.
6. González A, Ramírez MP, and Viadel V (2015). ICT Learning by Older Adults and their Attitudes toward Computer use. Curr Gerontol Geriatr Res. 2015:849308.
7. Fisch MJ, Chung AE, Accordino MK (2016). Using Technology to Improve Cancer Care: Social Media, Wearables, and Electronic Health Records. Am Soc Clin Oncol Educ Book. 35:200-8.
8. Greenhalgh T, A'Court C, Shaw S. MC Cardiovasc Disord. (2017). doi: 10.1186/s12872-017-0594-2. Understanding heart failure; explaining telehealth - a hermeneutic systematic review. Jun 14;17(1):156.
9. Kim, B. Y., & Lee, J. (2017). Smart Devices for Older Adults Managing Chronic Disease: A Scoping Review. JMIR mHealth and uHealth, 5(5), e69. http://doi.org/10.2196/mhealth.7141
10. Panahiazar M, Taslimitehrani V, Pereira N, Pathak J, (2015). Using EHRs and Machine Learning for Heart Failure Survival Analysis. “Stud Health Technol Inform”. 216:40-4.
